# Gender differences in cementless short stem total hip arthroplasty: significantly higher femoral lengthening in female patients

**DOI:** 10.1038/s41598-024-51621-7

**Published:** 2024-01-11

**Authors:** Christian Stadler, Sandra Feldler, Stella Stevoska, Clemens Schopper, Tobias Gotterbarm, Matthias Luger

**Affiliations:** 1 Department for Orthopaedics and Traumatology, Kepler University Hospital GmbH, Med Campus III, Krankenhausstr. 9, 4020 Linz, Austria; 2 Johannes Kepler University Linz, Altenberger Str. 96, 4040 Linz, Austria

**Keywords:** Bone, Orthopaedics

## Abstract

Modern cementless short stems in total hip arthroplasty (THA) enable a precise reconstruction of the native pre-arthritic hip geometry. While gender differences have been reported for older generation straight hip stems, there are hardly reports regarding modern cementless short hip stems. Therefore, we aimed to evaluate the influence of gender differences in hip anatomy in cementless short stem THA. A total of 207 patients (109 females, 98 males) with unilateral THA and absence of contralateral joint space narrowing (Kellgren-Lawrence grade ≤ 2) were included. Acetabular height and offset as well as femoral height and offset were measured on pre- and 3-months-postoperative anteroposterior X-rays of the pelvis and compared to the contralateral hip. Additionally, implant position was evaluated on the postoperative radiograph. In male patients, the loss of acetabular offset was significantly greater than in females (p = 0.012), leading to a compensatory increase in femoral offset (p = 0.041). Femoral height discrepancy was significantly higher in females (p < 0.001), accounting for an increased global hip height discrepancy (p < 0.001). The mean acetabular anteversion was significantly greater in female patients (p < 0.001). Female patients are at higher risk of femoral lengthening in THA with a cementless short stem potentially caused by a further proximally conducted femoral neck osteotomy and show significantly higher cup anteversion angles. Therefore, surgeons should take special care to the level of neck resection and implant positioning in female patients.

## Introduction

Adequate restoration of the native hip geometry in total hip arthroplasty (THA) is crucial for achieving good functional results and patient satisfaction^1,2^ as well as avoiding adverse events such as impingement^3,4^, abductor muscle weakness^5,6^, leg length discrepancy (LLD)^7,8^, dislocation^9^ and polyethylene wear^10^.

The Global Hip Offset (GHO) is an indirect measure for the lever arm of the hip. It is represented by the sum of the femoral offset (FO), which is defined as the distance between the center of rotation (COR) of the hip and proximal femoral shaft axis (FSA), and the acetabular offset (AO), which is defined as the distance from the COR to the acetabular teardrop figure^11,12^. A reduction of GHO negatively affects abductor muscle strength and gait kinematics due to inadequate lever arm reconstruction^2,6^. A decrease in femoral offset (FO) of 5 mm or more might lead to inferior patient related outcome measures^1^. Leg length is another crucial parameter after THA as many authors recommend keeping LLD at a minimum (± 5 mm) to avoid functional deficiencies and residual pain after THA, although there is no clear consensus on a clinically relevant cut-off value regarding LLD^2,13,14^.

In order to restore GHO and leg length anatomically, morphologic differences in male and female hip anatomy must be considered in THA. Females on average have a smaller femur and femoral head with a smaller distance between lesser trochanter and femoral head center, which seems to tendentially lead to a further proximally conducted femoral neck cut, resulting in a greater residual femoral neck with increased risk of limb lengthening^15^. Males have a more medially located acetabulum with less anteversion and greater acetabular floor depth than females, potentially leading to a greater loss of acetabular offset (AO) if reaming down to the true acetabular floor is performed, which possibly limits patient satisfaction after THA^15,16^.

Gender-specific differences regarding hip anatomy have been reported to influence restoration of native hip geometry when performing THA using older generation straight stems^15^. In recent years, cementless short stems have been introduced to the market in order to allow a more accurate restoration of the hip geometry^17–19^. However, conclusive data regarding gender-specific differences in reconstructing femoral and acetabular anatomy in cementless short stem THA is rare. Therefore, we aimed to evaluate discrepancies between males and females after THA using a cementless short stem implant and hypothesized to find gender differences regarding the restoration of the native joint geometry.

## Materials and methods

### Study population

**Figure 1:**
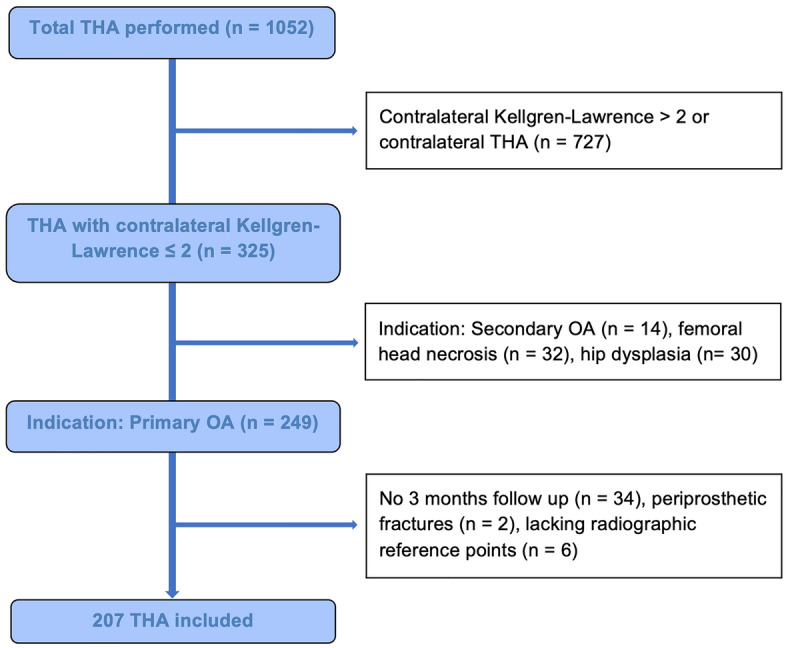
Shows the study design and formation of the study population.

This is a retrospective radiographical comparative study. A consecutive series of 1052 hips in 982 patients with index surgery between 2014 and 2019 were screened for inclusion and the medical records until 90 days postoperative were evaluated. In all cases the same short curved stem (Fitmore^®^, ZimmerBiomet, Warsaw, IN, USA) and bi-hemispherical press-fit acetabular cup (Allofit^®^/-S, ZimmerBiomet) were implanted via a minimally invasive supine anterolateral approach. Fitmore^®^ hip stem is a titanium alloy stem (Ti Al6V4) that has a porolock Ti-VPS coating in the proximal part to enhance bone ingrowth and is available in four different neck angle options (127°, 129°, 137°, 140°) and 14 different sizes (size 1–14) for each offset option^20^. The cementless titanium Allofit^®^/-S press-fit cup was implanted with or without screws. The preoperative X-rays of the pelvis (both hips in comparison, anterior–posterior view, standing upright) were screened for unilateral primary osteoarthritis (OA) of the hip. Patients with other diagnoses like dysplasia of the hip, avascular necrosis of the femoral head, posttraumatic OA or secondary OA due to systemic diseases such as rheumatism were excluded from this study. Further exclusion criteria were defined as bilateral OA of the hip (Kellgren Lawrence grade > 2), history of previous hip surgery, postoperative complication, reoperation or revision for any reason as well as missing pre- or postoperative radiographs^21^. A total of 207 patients met the inclusion criteria (Fig. 1).

Radiographic measurements were performed on preoperative and 3 months postoperative low centered anteroposterior (AP) radiographs of the pelvis in both groups. Preoperative age at operation, gender, body mass index (BMI) and laterality were recorded.

The study was approved by the ethics committee of the medical faculty of the Johannes Kepler University Linz (Reference number: 1239/2019). Due to the retrospective study design with evaluation of pre-existing medical records, the need for informed consent was waived by the ethics committee of the medical faculty of the Johannes Kepler University Linz. All procedures performed in studies involving human participants were in accordance with the ethical standards of the institutional and/or national research committee and with the 1964 Helsinki declaration and its later amendments or comparable ethical standards.

### Surgical technique and postoperative treatment protocol

The standardized peri- and postoperative protocol was identical in all cases. Surgical procedures were performed by surgeons with different levels of experience. At the study center, every attending performs at least 50 arthroplasties per year while residents are guided and supervised by an attending when performing surgery. In all cases a minimally invasive anterolateral Watson–Jones approach was performed. Fluoroscopy was not routinely used intraoperatively. Weight-bearing was tolerated immediately after surgery.

### Radiographic evaluation

**Figure 2:**
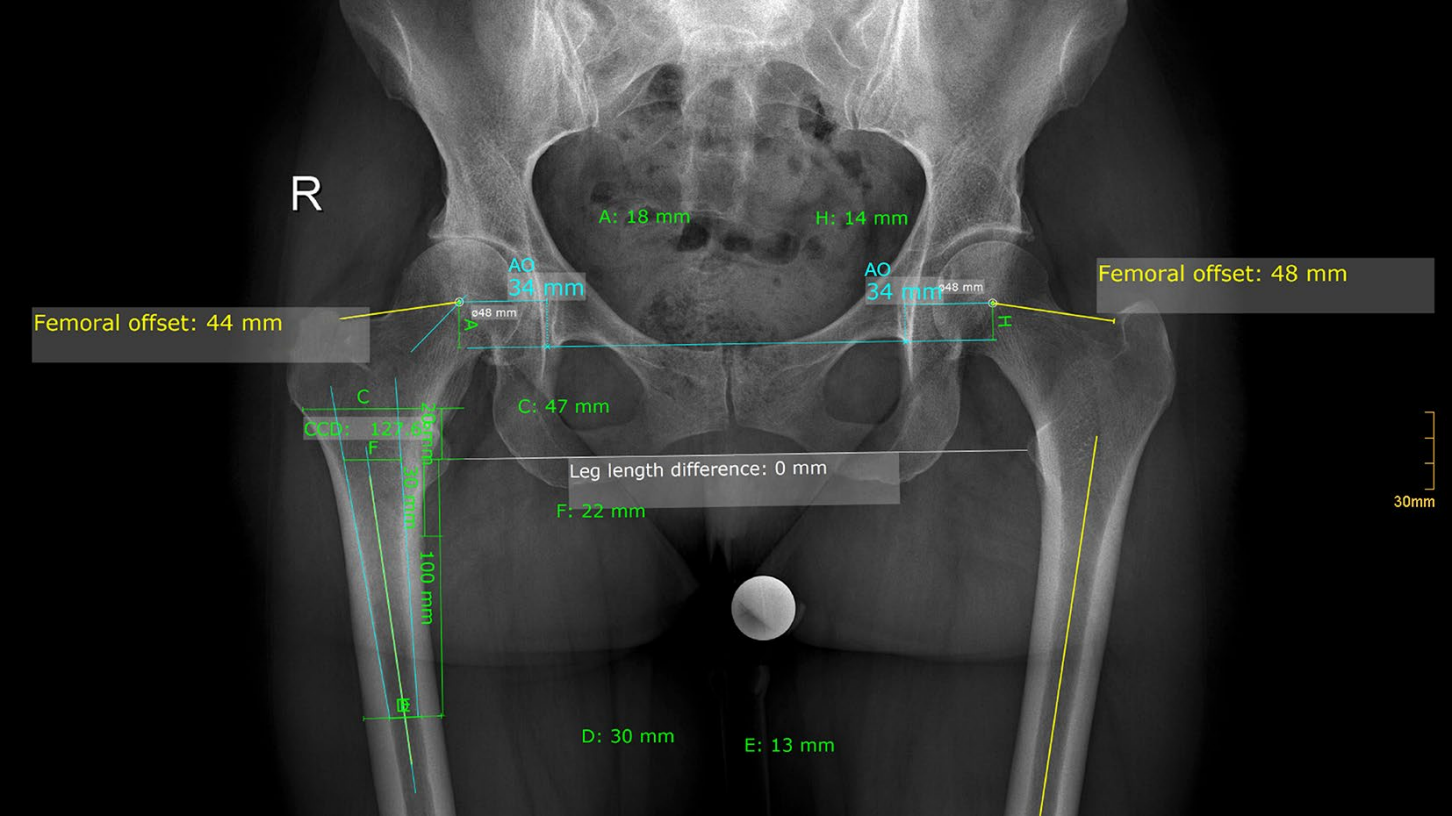
Shows the preoperative measurements. Measured values: both sides: femoral offset (FO), acetabular offset (AO), leg length difference; affected hip: centrum-collum-diaphyseal-angle (CCD-angle), cortical index (= (D − E)/D), canal flare index (= C/E), canal to calcar ratio (= E/F).

Radiographic measurements were performed on preoperative and 3 months postoperative digital low-centered AP radiographs of the pelvis independently by two reviewers (S.F., C.S.) who were not involved in the index surgery^11^. Radiographs were taken with the patient in standing position and both legs in 15° internal rotation with marking lines on the floor the ensure an equal standing position for each radiograph and the central beam directed on the symphysis pubis with a standardized film to focus distance of 1.15 m^22^. A double coordinate system was applied on both the preoperative and the postoperative images and calibration of the magnification factor was performed using a standardized metallic radiopaque ball with 25 mm diameter placed in a standardized position between the patients’ thighs to achieve accurate measurements of the hip anatomy^23,24^. MediCAD^®^ Software V5.1 (Hectec GmbH, Germany) was used to perform the radiographic analysis^25^. The hip center of rotation (COR) was defined using a circle tool determining the diameter of the femoral head and its center^12^. The femoral offset (FO) was determined as the perpendicular distance between the COR and the FSA^11,12^. AO was measured as the perpendicular distance between the COR and line T, with T being the perpendicular line on the transteardrop line (TT) through the ipsilateral teardrop figure^11^. Global Hip offset (GHO) was calculated as the sum of FO and AO^11^. Acetabular height discrepancy (AHD) was measured as the differences between the perpendicular distance of the COR to line TT between the operated and non-affect contralateral side^15,17^. Femoral height discrepancy (FHD) was measured as the difference of the perpendicular distance between line TT and the middle of the lesser trochanter (LT) between the operated and non-affect contralateral side^15,22^. The addition of the acetabular height discrepancy and the femoral height discrepancy provided the overall global height discrepancy (GHD) with a negative number reflecting shortening and a positive number lengthening^15^. Centrum-Collum-Diaphyseal (CCD) angle was determined according to M. E. Müller on the affected hip^26^. To characterize the anatomical shape of the proximal femur and the thickness of cortical bone, the canal to calcar isthmus ratio and the cortical index (CI) according to Dorr et al.^27^ were determined. A high CI indicates a thick cortical bone^27^. Additionally the canal flare according to Noble et al.^28^ was determined. The stem alignment was measured as the difference in degrees between the anatomic femoral shaft axis and the vertical stem axis^29^. On preoperative X-rays FO, AO and GHO were measured bilaterally. Acetabular height discrepancy (AHD), femoral height discrepancy (FHD) and global height discrepancy (GHD) between the osteoarthritic and the contralateral side were analyzed. CCD-angle, CI, Canal Flare Index and Canal to Calcar Ratio were measured unilaterally on the affected hip (Fig. 2).

**Figure 3:**
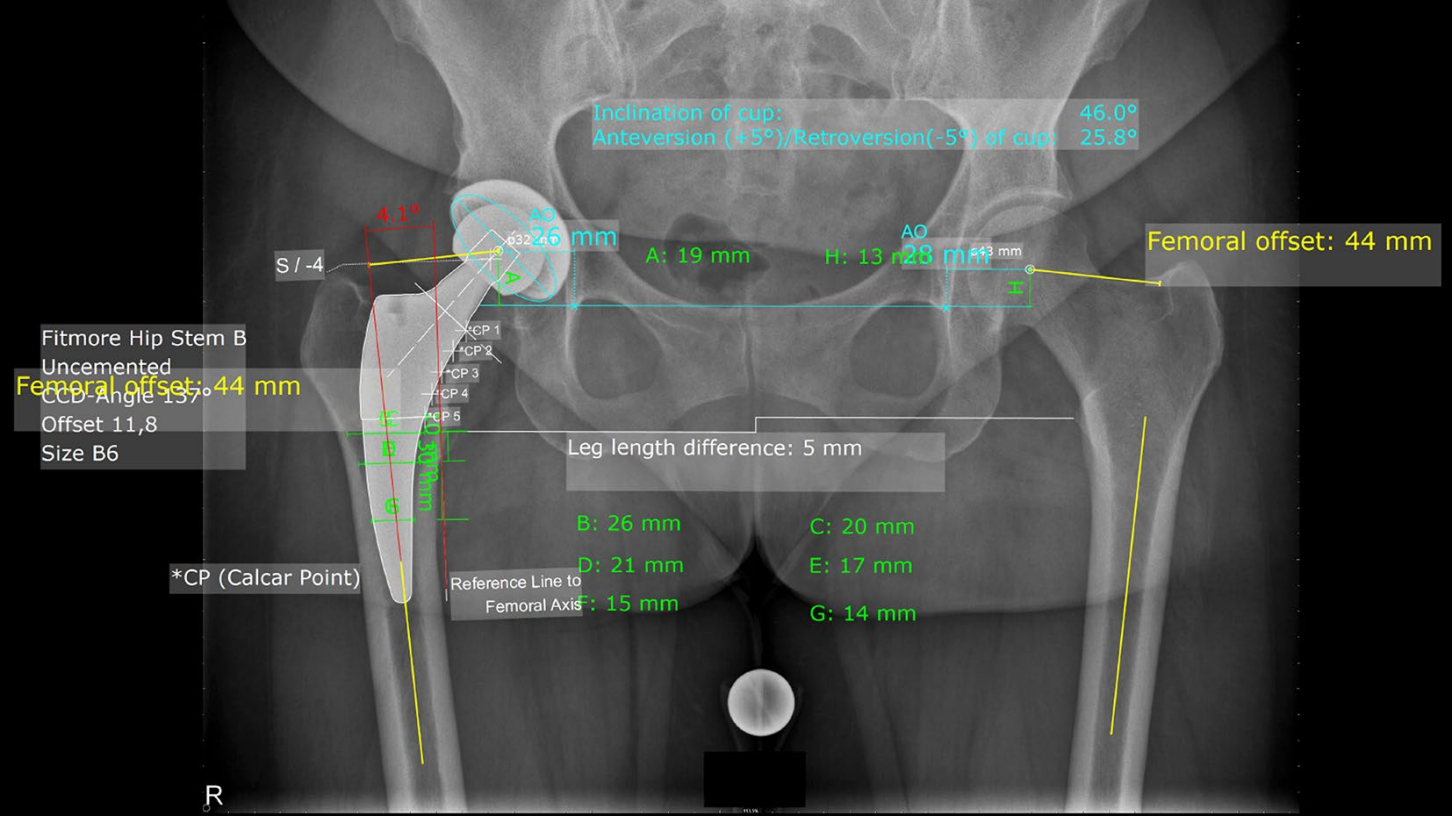
Shows the postoperative measurements. Measured values: both sides: femoral offset (FO), acetabular offset (AO), leg length difference. Affected hip: cup inclination, cup anteversion, stem alignment, canal fill index I (C/B), II (E/D) and III (G/F).

On postoperative X-rays FO, AO and GHO were measured bilaterally. Postoperative AHD, FHD and GHD between the operated and the contralateral side were analyzed, while cup inclination, cup anteversion, stem alignment and canal fill indices I–III were measured unilaterally on the operated hip (Fig. 3).

### Statistical analysis

Statistical analysis was performed using SPSS version 28 (IBM SPSS statistics, Chicago, IL, USA). Arithmetic mean value and standard deviation were calculated for metric scaled data. Shapiro–Wilk-test was performed to test for normal distribution. All evaluated parameters were normally distributed. Chi-square-test was performed to analyze categorial parameters while *t*-test was performed to analyze metric scaled parameters. Intra- and interobserver reliabilities were calculated. Intra-class-correlation coefficients (ICC) were used with a two-way random effects model for absolute agreement. Repeated measurements for intra-observer reliability were conducted at a time interval of two weeks in a blinded fashion.

A p value < 0.05 was considered statistically significant.

## Results

A total of 207 patients were included in this study with 52.7% of the study population being female patients (Table 1). Intra-observer ICC between the 2 sets of measurements was 0.94 (95% CI 0.90–0.98, *p* < 0.001) while inter-observer ICC was 0.90 (95% CI 0.85–0.95; *p* < 0.001).

**Table 1:** Shows the patient demographics of the study population and the surgeon’s experience.

Variable	Female	Male	*p* value
Number of Patients	109 (52.7%)	98 (47.3%)	–
Age at operation (years)	64.2 ± 10.3	63.7 ± 10	0.766
BMI (kg/m^2^)	28.1 ± 5.3	29.6 ± 4.8	**0.033**
Height (cm)	164.1 ± 6.5	175.7 ± 6.9	**< 0.001**
Weight (kg)	75.5 ± 14.1	91.5 ± 15.6	**< 0.001**
Side (left:right)	50:59	42:56	0.663
Kellgren Lawrence			0.315
Grade 1	45 (41.5%)	33 (33.7%)	
Grade 2	64 (58.7%)	65 (66.3%)	
Surgeon’s experience			0.803
Attending	75 (70.4%)	69 (68.8%)	
Resident	34 (29.6%)	29 (31.2%)	

Significant values are in bold.

Preoperative measures revealed a significant greater FO, AO and GHO in males at the operated side as well as at the contralateral side compared to females (Table 2).

**Table 2:** Shows the results of the preoperative radiographic measurements.

Radiographic measure	Female	Male	*p* value
FO operated side (mm)	38.0 ± 6.3	42.6 ± 6.2	**< 0.001**
FO contralateral side (mm)	40.6 ± 5.6	45.1 ± 6.6	**< 0.001**
FO difference (mm)	− 2.6 ± 4.1	− 2.5 ± 4.3	0.850
AO operated side (mm)	32.6 ± 3.4	36.7 ± 5.5	**< 0.001**
AO contralateral side (mm)	31.2 ± 4.1	35.7 ± 3.4	**< 0.001**
AO difference (mm)	1.4 ± 4.2	1.0 ± 4.3	0.442
GHO operated side (mm)	70.6 ± 6.6	79.3 ± 8.2	**< 0.001**
GHO contralateral side (mm)	71.8 ± 6.3	80.9 ± 8.0	**< 0.001**
GHO difference (mm)	− 1.2 ± 4.8	− 1.6 ± 5.9	0.648
AHD (mm)	2.5 ± 4.2	2.4 ± 4.0	0.429
FHD (mm)	− 2.4 ± 5.5	− 3.4 ± 6.0	0.114
GHD (mm)	0.1 ± 4.1	− 1.0 ± 4.8	**0.041**
CCD angle (degrees)	129.9 ± 11.5	128.6 ± 5.6	0.315
Cortical Index	0.6 ± 0.1	0.6 ± 0.2	0.954
Canal Flare Index	4.1 ± 0.8	4.0 ± 0.9	0.601
Canal to Calcar ratio	0.6 ± 0.2	0.6 ± 0.1	0.185

*FO* femoral offset, *AO* acetabular offset, *GHO* global hip offset, *AHD* acetabular height discrepancy, *FHD* femoral height discrepancy, *GHD* global hip discrepancy. Significant values are in bold.

Evaluation of the postoperative radiographs revealed significantly greater overall FO, AO and GHO in male patients. There was a significantly higher gain in FO as well as a significantly greater loss in AO after THA compared to the contralateral side in male patients. There was no significant gender specific difference regarding AHD between the operated and the contralateral side. FHD as well as GHD between operated and contralateral side as well as cup anteversion were significantly higher within the female study population (Table 3).

**Table 3:** Shows the results of the postoperative radiographic measurements.

Radiographic measure	Female	Male	*p* value
FO operated side (mm)	44.0 ± 6.5	50.5 ± 7.2	**< 0.001**
FO contralateral side (mm)	40.0 ± 5.7	44.4 ± 6.4	**< 0.001**
FO difference (mm)	4.2 ± 6.0	6.0 ± 6.9	**0.041**
AO operated side (mm)	28.4 ± 2.9	31.7 ± 3.7	**< 0.001**
AO contralateral side (mm)	31.0 ± 3.1	35.8 ± 4.7	**< 0.001**
AO difference (mm)	− 2.5 ± 3.7	− 4.1 ± 5.1	**0.012**
GHO operated side (mm)	72.4 ± 6.6	82.2 ± 7.6	**< 0.001**
GHO contralateral side (mm)	70.7 ± 6.7	80.2 ± 8.4	**< 0.001**
GHO difference (mm)	1.7 ± 5.9	2.0 ± 6.9	0.740
AHD (mm)	4.0 ± 4.0	4.6 ± 4.0	0.118
FHD (mm)	1.4 ± 5.6	− 2.3 ± 6.1	**< 0.001**
GHD (mm)	5.4 ± 5.3	2.4 ± 5.6	**< 0.001**
Cup Inclination (degrees)	44.9 ± 6.1	43.6 ± 5.9	0.121
Cup anteversion (degrees)	30.3 ± 6.1	24.5 ± 6.8	**< 0.001**
Stem alignment (degrees)	4.2 ± 2.8	5.0 ± 3.1	0.069
Canal Fill Index I	0.8 ± 0.1	0.8 ± 0.1	0.285
Canal Fill Index II	0.8 ± 0.1	0.8 ± 0.1	0.983
Canal Fill Index III	0.9 ± 0.4	0.9 ± 0.1	0.805

*FO* femoral offset, *AO* acetabular offset, *GHO *global hip offset, *AHD* acetabular height discrepancy, *FHD* femoral height discrepancy, *GHD* global hip discrepancy. Significant values are in bold.

## Discussion

The results of this study reveal significant gender specific differences regarding the preoperative anatomical hip geometry as well as the postoperative changes after cementless short stem THA in various measures.

Male patients had a significantly higher preoperative FO, AO and GHO compared to female patients (p < 0.001). Postoperatively, the loss of AO was significantly higher in male than in female patients (p = 0.012). These findings match the reports of other authors and might be attributed to the greater acetabular floor depth in male patients resulting in an increased loss of AO when reaming down to the true acetabular floor is performed^15,16,30^.

This study revealed a significantly higher postoperative FO difference compared to the contralateral hip in males than in females, as an increase in FO was presumably required to compensate the loss of AO. However, no significant gender-specific discrepancy regarding GHO was detected with an average GHO difference < 5 mm compared to the contralateral hip in both groups. Previous studies reported a 5 mm cut-off-value for global offset, as a discrepancy of GHO exceeding this value seems to negatively affect functional outcomes and gait kinematics, owing to an inadequate reconstruction of the lever arm and consecutive abductor muscle weakness^1,6,31^. A GHO discrepancy > 10 mm compared to the native hip was also demonstrated to limit the improvement in Oxford Hip Score at one year postoperatively^32^. Additionally, excessive changes in AO seem to be associated with worse clinical outcomes even if an accurate reconstruction of GHO was obtained as changes of the AO might affect the range of motion due to bony impingement^3,33^. Furthermore, an increase in FO exceeding 5 mm is reported to accelerate polyethylene wear^10^. Moreover, patients with lower FO seem to tendentially report less pain postoperatively when compared to patients with higher FO^15,33^. While postoperative changes of ± 5 mm regarding the FO seem to affect the outcome after THA, it remains questionable whether the relatively small average difference between females and males in postoperative FO of 1.8 mm found in this study actually has clinical relevance regarding the functional outcome^2^. While in the present study, the GHD increased in both females and males postoperatively, females showed significantly higher postoperative FHD and subsequently also a significantly higher GHD than males. An increase in global hip height accounts for lengthening of the affected limb^15,32^. Females usually have a smaller femur and femoral head than males, which might lead to bias towards a more proximal femoral neck cut with an increase in femoral height, which might lead to a reduced improvement in pain after THA^15^. Although data regarding the effect of LLD after THA are inconsistent and other factors than global hip height such as knee alignment and pelvic obliquity contribute to patient-perception of LLD^34^, an inadequate restoration of hip height seems to negatively affect clinical outcomes^2,13–15,35^. Patients with > 10 mm global height discrepancy were demonstrated to experience residual pain after one year postoperatively^32^.

Postoperative acetabular anteversion was significantly greater in female patients than in males (p < 0.001), which might reflect the greater anteversion of the native female acetabulum reported by some authors^36,37^. Cup anteversion and inclination after THA are of special interest, as they are considered as key-factors to avoid dislocation^38,39^. Some authors recommend safe-zones for cup positioning (anteversion 15° ± 10°, inclination 40° ± 10°) to prevent dislocation^39^. While cup inclination of male and female patients was within the recommended safe-zones mentioned above, the average cup anteversion of female patients was 30.3° ± 6.1°, which is out of the recommended safe-zone at the present study^39^. One factor, that might have led to cup positioning out of the safe zone was the lack of intraoperative fluoroscopy^40^. Due to the study’s design it’s not possible to comment on potential impacts of these anteversion values in female patients on the dislocation rates. However, a previous study investigating the same implant and minimally invasive anterolateral approach reported a rate of dislocations of 1% within the first 90 days after surgery^41^. Besides, several authors suggest that the combination of restoration of the native center of rotation, soft tissue tension and avoidance of impingement have greater effect on the risk of dislocation than cup positioning alone^9,42–44^. While a high risk of cup malpositioning using a minimally invasive anterolateral approach is reported^22^, the higher cup anteversion in female patients might have tendentially led to instabilities which possibly have been addressed intraoperatively by performing femoral lengthening in order to generate a stable overall implant composition.

The results of this study must be interpreted with respect to several limitations, which are mainly caused by the retrospective study design. Patient reported outcome measures were not collected routinely as part of the follow-up after THA. While in general male patients seem to tendentially show better clinical outcomes with better functional status after THA, no evaluation of the patient satisfaction and the actual clinical effect of the results of this study was possible^45,46^. Also, within this study no postoperative complications were analyzed as patients with intra- or postoperative complications and consecutive revisional surgeries were excluded from this study. Therefore, no conclusions regarding the possible effects on the complication rate of this study’s findings can be drawn. Additionally, the measurements were conducted on anteroposterior radiographs of the pelvis, which are reported to be susceptible for projection errors including for example an underestimation of the FO of about 13% compared to measurements taken on CT-scans, which represents a major limitation of this study^12,47^. However, the aim of the study was to evaluate relative changes to the hip geometry compared to the contralateral hip rather than determining absolute values. Additionally, computed tomography (CT) of the hip is not part of the routinely performed management of THA at the study center due to increased radiation exposure and cost factors.

In conclusion, female patients are at higher risk of femoral lengthening in THA with a cementless short stem with multiple offset options. Additionally, acetabular cups are placed with significantly higher anteversion angles and more often outside the safe zone in female patients. Therefore, surgeons should take special care to the level of neck resection and implant positioning in female patients.

## Data Availability

The datasets used and analyzed during the current study are available from the corresponding author on reasonable request.
